# Global health ethics: critical reflections on the contours of an emerging field, 1977–2015

**DOI:** 10.1186/s12910-019-0391-9

**Published:** 2019-07-25

**Authors:** Gail Robson, Nathan Gibson, Alison Thompson, Solomon Benatar, Avram Denburg

**Affiliations:** 1 Joint Centre for Bioethics, University of Toronto, Toronto, Canada; 2 Leslie Dan Faculty of Pharmacy, University of Toronto, Toronto, Canada; 3 Faculty Of Health Sciences, University of Cape Town, Cape Town, South Africa; 4 Dalla Lana School of Public Health, University of Toronto, Toronto, Canada; 5 Department of Paediatrics, University of Toronto, Toronto, Canada; 6 Institute of Health Policy, Management, and Evaluation, University of Toronto, Toronto, Canada; 7 Division of Haematology/Oncology, The Hospital for Sick Children, 555 University Ave, Toronto, Ontario M5G 1X8 Canada

**Keywords:** Global health, Ethics, Critical interpretive review

## Abstract

**Background:**

The field of bioethics has evolved over the past half-century, incorporating new domains of inquiry that signal developments in health research, clinical practice, public health in its broadest sense and more recently sensitivity to the interdependence of global health and the environment. These extensions of the reach of bioethics are a welcome response to the growth of global health as a field of vital interest and activity.

**Methods:**

This paper provides a critical interpretive review of how the term “global health ethics” has been used and defined in the literature to date to identify ethical issues that arise and need to be addressed when deliberating on and working to improve the discourse on ethical issues in health globally.

**Results:**

Selected publications were analyzed by year of publication and geographical distribution, journal and field, level of engagement, and ethical framework. Of the literature selected, 151 articles (88%) were written by authors in high-income countries (HIC), as defined by the World Bank country classifications, 8 articles (5%) were written by authors in low- or middle-income countries (LMIC), and 13 articles (7%) were collaborations between authors in HIC and LMIC. All of the articles selected except one from 1977 were published after 1998. Literature on global health ethics spiked considerably from the early 2000s, with the highest number in 2011. One hundred twenty-seven articles identified were published in academic journals, 1 document was an official training document, and 44 were chapters in published books. The dominant journals were the American Journal of Bioethics (*n* = 10), Developing World Bioethics (*n* = 9), and Bioethics (*n* = 7). We coded the articles by level of engagement within the ethical domain at different levels: (1) interpersonal, (2) institutional, (3) international, and (4) structural. The ethical frameworks at use corresponded to four functional categories: those examining practical or narrowly applied ethical questions; those concerned with normative ethics; those examining an issue through a single philosophical tradition; and those comparing and contrasting insights from multiple ethical frameworks.

**Conclusions:**

This critical interpretive review is intended to delineate the current contours and revitalize the conversation around the future charge of global health ethics scholarship.

**Electronic supplementary material:**

The online version of this article (10.1186/s12910-019-0391-9) contains supplementary material, which is available to authorized users.

## Background

The field of bioethics has evolved considerably over the past half-century, incorporating new domains of inquiry that mirror developments in health research, clinical practice, public health in its broadest sense and more recently insights into the interdependence of global health with the environment. From its roots in medical and research ethics, bioethics scholarship has thus diversified into the ethics of public health and health policy. More recently, expansion of global health as a field of special interest has led to consideration of the ethical dilemmas that arise and need to be addressed when deliberating on improving health globally in a world divided by wide (and widening disparities) in health [1]. However, the definition and purview of ‘global health ethics’ is incompletely understood or developed. Few attempts have been made to review the “contours the field” [2] from a critical perspective; indeed, its status as a distinct field remains uncertain and contested.

In this paper, we report the results of a critical interpretive review [2] on the emerging literature in ‘global health ethics’, which seeks to map the terrain of this evolving field, and understand its relationship to established disciplines and domains of inquiry in bioethics. Our aim is not to drive towards a definition of global health ethics but rather to understand its various current conceptualizations in the academic literature and critically review these findings. Through a descriptive review of the body of work that explicitly identifies as ‘global health ethics,’ we aim to outline the present contours of the field, in the hopes of informing debate on its centre, boundaries, limitations and future prospects. We provide some critical insights into how this debate can be moved forward ethically.

## Methods

The aim of this paper was to provide a Critical Interpretive Review of the literature. We chose this methodology because our goal was not to review ‘evidence,’ as is often the goal of systematic reviews or even some scoping reviews. A Critical Interpretive Review best describes the methodological process we undertook to go beyond description and include a degree of analysis and conceptual clarification. Such a review does not aim to provide ‘an answer’, but rather seeks to provide analysis and synthesis [3]. Given the diversity of views on what constitutes global health ethics, we felt this kind of review provides an opportunity to “take stock” [3] of the literature to date, and to provide a “launch pad” [3] for further conceptual debate and development on this important issue. We proceeded from the premise that discourse not only reflects ideas, but that it is productive and reproductive of things such as social arrangements and power dynamics, and is therefore of moral relevance to bioethical debate [4]. Questions such as ‘who can speak?’ and ‘where are the discursive fissures and tensions occurring and what can they tell us?’ are critical questions that we hope to attend to in this paper.

One of the main drawbacks of the methodology, however, is that it is often faulted for its lack of systematicity in its approach to reviewing literature [3, 5]. In order to improve the methodological rigour, transparency and rationale for our search strategy, we (somewhat unusually) employed a modified version of Arksey & O’Malley’s six-stage methodological framework as expanded by Levac et al [6, 7]. However, the scoping review methodology outlined below was a means to an end, rather than an end in itself. The six stages from the original methodology are: (1) identify the research question, (2) search for relevant studies, (3) select studies, (4) chart the data, (5) collate, summarize, and report the results, and (6) an optional consultation process with stakeholders [6]. We chose to limit the scope of this paper to the first 5 framework steps. While we did meet with stakeholders to inform the outline of our paper and develop criteria for charting the data and study selection, we did not undertake stakeholder consultation to direct knowledge translation. Given that this study aims to present how ‘global health ethics’ is defined in existing literature, and to provide critical reflections on our findings to spur further debate, we omitted this last step from their methodology.

### Research question

Our aim in this review was not to come up with a definition of ‘global health ethics’ or to decide what articles *should* be considered under that umbrella, but rather to investigate how this term is being used and defined in the literature. Therefore, we left the research question open-ended and kept the search terms broad in order to be as inclusive as possible of all literature using these terms. Our overarching question was: *How is ‘global health ethics’ defined by those using the term or related terms in the literature?* We did not apply our own definition of ‘global health’, ‘ethics’ or the combination ‘global health ethics’. We used the research question to explore the context in which these terms are being explicitly employed to map the scope of the field and determine whether there are any emergent trends.

### Identify relevant studies and study selection

We used the MESH terms ‘global health’ and ‘ethic*’ to search in Embase, Medline, Philosopher’s Index and Scopus databases. These four databases were chosen with the assistance of a research librarian to allow for a comprehensive search of both social science, philosophical literature and medical, health-focused literature. The searches were exploded to include all subheadings available in each database (including keywords) and then limited to articles that were written in English. Embase identified 561 possibly relevant articles, while Medline identified 1289, Scopus identified 2613, and Philosopher’s Index identified 158. In addition to systematic searches, we conducted purposive sampling within the systematically derived sample by examining references in all review-style papers along with a random sample of papers within the main sample in order to identify other relevant articles being cited within the systematically searched for articles. This form of snowball sampling, commonly used in scoping reviews helps to ensure that articles that fall within the scope of the review but that are not captured by systematic searches are not omitted from the sample [8]. Duplicate articles found in multiple databases were removed. Article abstracts were reviewed by two members of the research team (GR, NG) to verify that they self-identified as ‘global health ethics’ through their use of keywords, description in the abstract and/or title wording. Both grey literature and academic articles were considered. Grey literature included guideline documents and editorials, but the majority of the articles included were theoretical and conceptual academic articles with some empirical studies. The review also includes books, which were obtained from the University of Toronto library system or as e-books. Chapters written by different authors within one book are considered separate articles in our analysis. The relevant articles were extracted from the databases and compiled into a list on the reference management software *Zotero*. One hundred eighty-six articles were identified as relevant in the first round and then reviewed by the two main authors separately to further determine whether or not they were relevant. The articles were judged based on each article’s keywords and title, as well as content in so far as the presence of the words ‘global health ethics’ were present in some form, or the article discussed these issues in different, but related terms. We would exclude, for example, an article that came up with our search terms analyzing the intersection of ethics and the social determinants of health if the article was solely analyzing a case study in one location and did not itself use the term ‘global health’ or have a substantial analysis of the ‘global’ connection*.* Those articles that were marked as possibly irrelevant to the study went through the remaining authors and relevance was determined on an individual basis using the cumulative judgement of all authors. We included all articles that appeared with our search strategy and did not exclude any articles by date published. The publication date of the articles included ranged from 1977 to 2015. The final list included 172 articles. The search process was completed during summer 2015.

### Assessment and analysis

The final list of 172 identified articles was read by two authors (GR, NG) and information was extracted from each article based on charting criteria, including: year published, geography of authors, journal, academic field, and methodology (see Table 1).

**Table 1:** Charting Criteria

1.	Date of publication
2.	Geography by country of authors
3.	Level of scope: (1 – interpersonal, 2 – institutional, 3 – international, 4 – structural)
4.	Journal
5	Academic Field (public health, philosophy, medical anthropology, etc.)
6.	Methodology
7	‘Global Health’ definition
8	Ethical framework
9.	Specific issue addressed

The articles selected covered a broad range of topics under the umbrella of ‘global health’, ranging from procedural medical ethics to ethical implications of global environmental degradation and climate change for health. Through consultation with all authors, supplemented by input from bioethics and global health experts, a system was created to code articles into four categories: (1) interpersonal, (2) institutional, (3) international, and (4) structural. These categories were created to aid in analysis and understand what level of interaction was being analyzed under the heading of ‘global health ethics’. This was a form of deductive, descriptive coding that was based on the notion that at its heart, ethics is about relationships and the obligations we have to one another. These codes are an attempt to categorize these data on the basis of who the moral actors are in the context of relationships being described and argued for in the data set. This is important because much of the global health ethics literature is an attempt to argue for particular sets of obligations by particular moral agents, whether they are medical students, researchers, NGOs, nation states, or international actors. Being able to capture this information, and to map the contours of these moral relationships in the discourse on global health ethics was essential to our analysis. While some of the papers covered in this review would be coded as more than one level, papers tended to be focused at one end of the spectrum of relationships than the other, i.e. it was very rare that authors would focus on both political economy and individual relations between actors in the context of a medical encounter. This becomes important as we begin to map the contours of the debate over what counts as a legitimate level of focus for the field of global health ethics. This fits with the methodological aim of the paper to view the literature “as an object of scrutiny in its own right” [9] with the aim of “identifying normative issues arising from the study” which entails additional critical scrutiny [2].

The first category—interpersonal ethics—referred mainly to doctor-patient, or researcher-subject relationships within a global context. The second category—institutional—encompassed institutional ethics related to hospitals, medical systems, or academic institutions, examined with respect to their respective roles and duties in health provision, and attendant ethical issues in differing contexts. Our categorical distinction between ‘international’ and ‘structural’ centered principally on the locus of authority for decision-making, and the resulting corollary duties. Articles in the ‘international’ category examined the role of the government, institutions and individuals of a given country in their biomedical relationships with other countries – for example, the role and duties of the Canadian government in provision of health aid in international humanitarian contexts, and the role of institutions (e.g. the Hospital for Sick Children in Toronto) and individuals from such institutions in taking on collaborative work with institutions and colleagues abroad. Those in the ‘structural’ category, by contrast, considered factors beyond the realm of national governments, including environmental health and climate change, the role of macroeconomic conditions and global financial regimes on population health, as well as normative inquiry into the rights of populations. We extracted information from each article on: (1) its definition of global health, (2) the ethical framework used, and (3) the specific issue discussed. For example, a relevant article might: (1) define global health as a collaborative research enterprise between low- and high-income countries and (2) use a global justice framework to (3) look specifically at HIV research in a particular country.

## Results

### Year of publication and geographical distribution

**Fig. 1:**
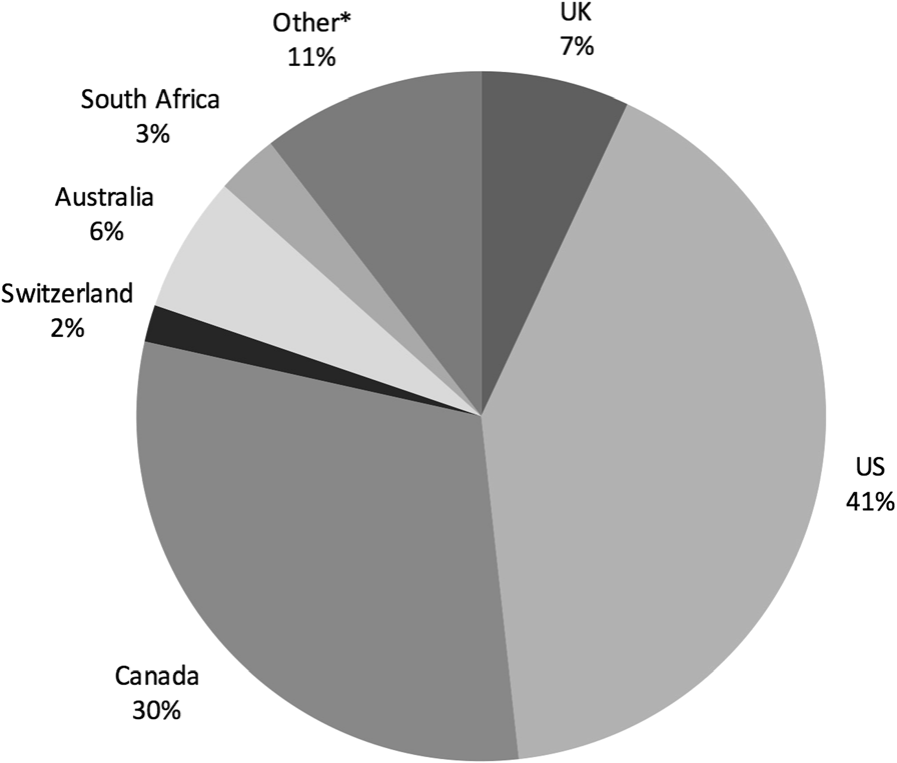
Geography by country of authors

**Fig. 2:**
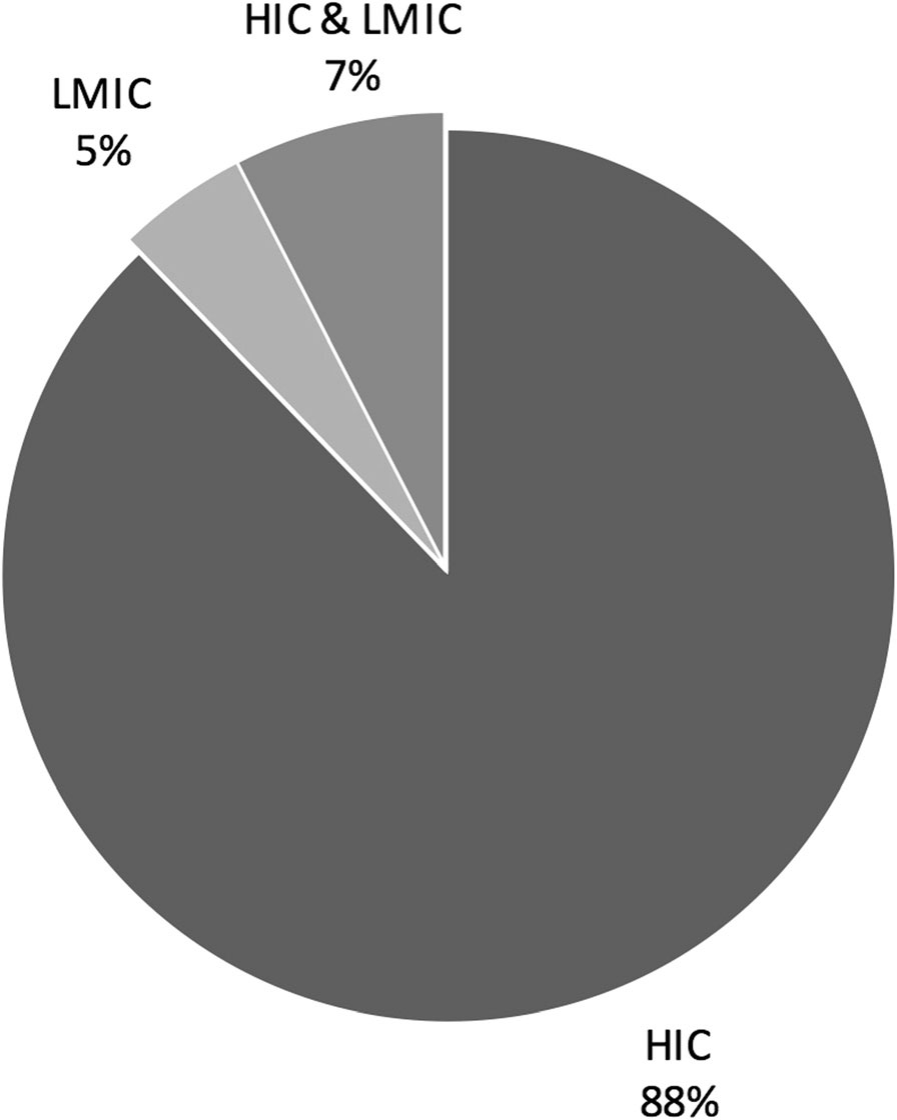
Economic classification by country author

We determined the geographical distribution of articles by country of the authors or the location of the principal research institution at which the research was done. As shown in Fig. 1*: Geography by country of authors*, the majority of the articles were from North American institutions. As shown in Fig. 2*: Economic classification of author country*, of the literature selected, 151 articles (88%) were written by authors in high-income countries (HIC), as defined by the World Bank country classifications, 8 articles (5%) were written by authors in low- or middle-income countries (LMIC), and 13 articles (7%) were collaborations between authors in HIC and LMIC. We found no articles that were collaborations between authors in multiple LMICs with no HIC involvement. Of the 13 collaborative articles, 5 listed the LMIC authors first and 8 listed HIC authors first. The bias towards high-income countries and North American institutions could be in part due to the English language restriction contained in our search strategy. The four top countries for first author – US, Canada, Australia and the UK – are all primarily English-speaking.

**Fig. 3:**
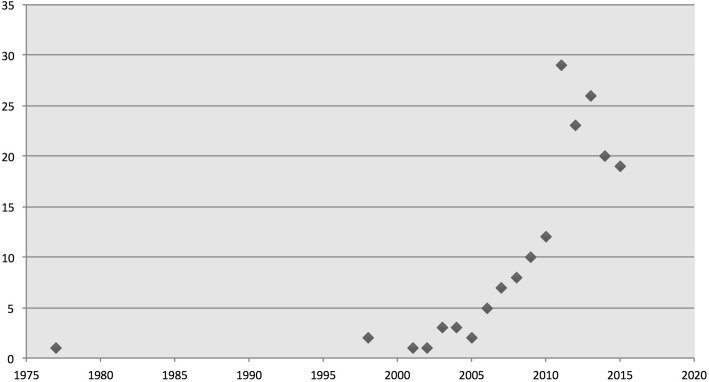
Temporal distribution of articles

We determined temporal distribution by using the date of publication for each article. All of the articles selected except one from 1977 were published after 1998. The 1977 article and literature from the late 1990s were more likely to use the term ‘international health’ and discuss the globalization of public health ethics or bioethics rather than discuss ‘global health ethics’ per se. However, a 1998 article addressed the exploitative nature of the global political economy and mentioned the ethical implications of environmental change on health [10]. A subsequent article explicated in more detail broader discourses on bioethics [1]. As seen in Fig. 3*: Temporal distribution of articles*, Literature on global health ethics spiked considerably from the early 2000s, with the highest number of articles (29) in 2011 (see Fig. 3) with 98% of the articles included published since 2000, and a multi-authored book ‘Global Health and Global Health Ethics’ published in 2011 [11].

### Journal and field

One hundred twenty-seven articles identified were published in academic journals, 1 document was an official training document, and 44 were chapters in published books. The predominant journals were the American Journal of Bioethics (*n* = 10), Developing World Bioethics (*n* = 9), and Bioethics (*n* = 7). The methodology of the articles included 5 case studies, 9 empirical research papers, all of which were qualitative, 5 literature reviews, one report from a conference, and 152 conceptual articles, of which 7 were editorials (see Table 2).

**Table 2:** Type of publication

Type of publication	n (%) of publications
Source	
Academic journal	127 (74%)
Training document	1 (1%)
Book Chapter	44 (26%)
Methodology	
Case studies	5 (3%)
Empirical research	9 (5%)
Literature review	5 (3%)
Conference report	1 (1%)
Conceptual	152 (88%)

**Fig. 4:**
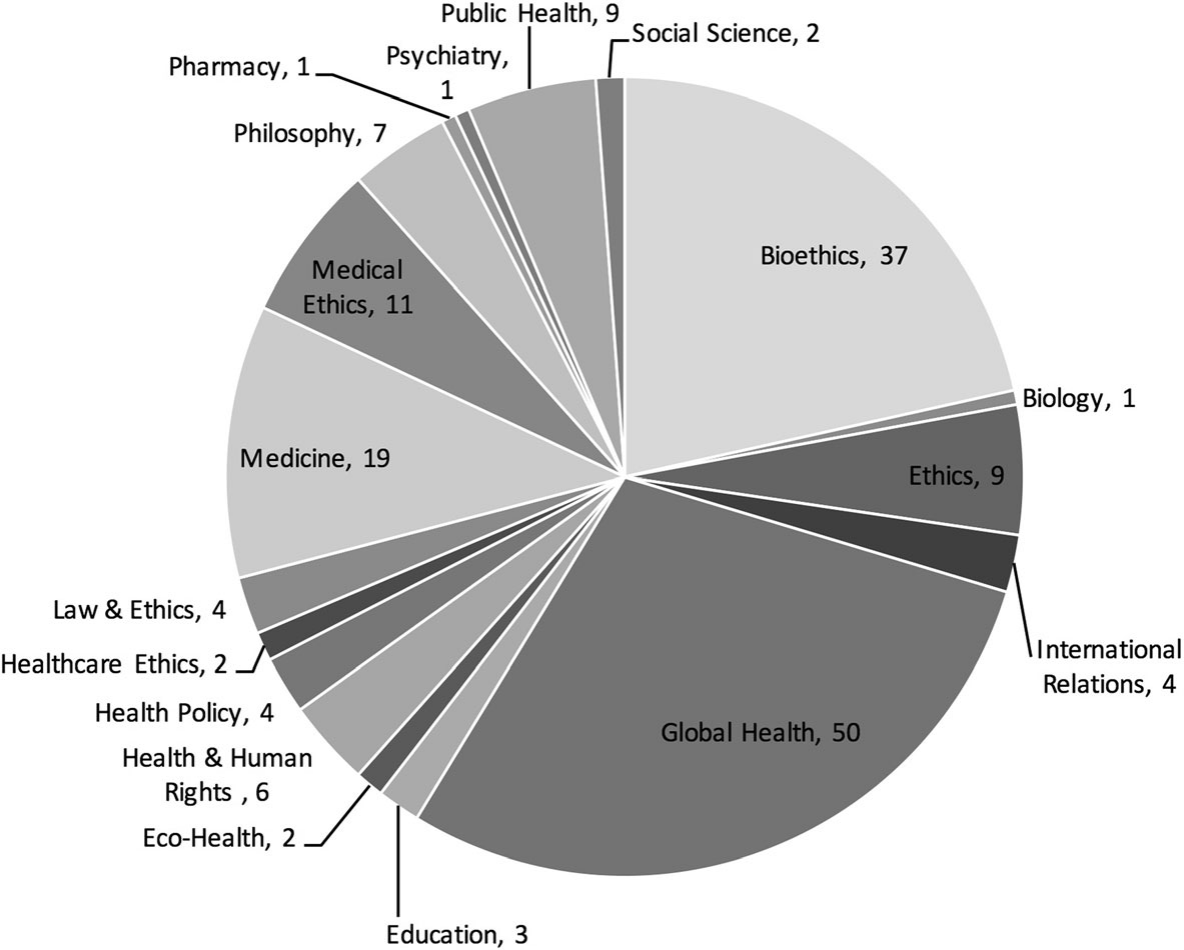
Articles by field

After dividing the journals and books by field, we found that the greatest number of articles came from journals and books specifically devoted to global health, with 36 book chapters and 14 journal articles, and to bioethics, with 37 journal articles (see Fig. 4*: Articles by field*). The breadth of other fields, however, demonstrated the wide disciplinary and content range that characterizes global health ethics scholarship. Journal fields included medicine, public health, psychiatry, education, international relations and law.

### Level of engagement

We coded the articles by level of engagement within global health into the four categories delineated above: (1) interpersonal, (2) institutional, (3) international, and (4) structural. Many articles did not fit neatly into a single category; each article was assigned to one or more categories.

#### Interpersonal

Twelve articles were coded as level 1 (interpersonal). These articles dealt with medical ethics in a global setting, including medical education ethics, humanitarian ethics, and clinical research ethics. These articles considered the role of the individual doctor or researcher—usually from high-income countries—and their interactions with patients or subjects in low-resource settings. This level included the guideline document and training handbook which both examined ethical dilemmas for medical students in global health settings. Another 22 articles were assigned to both level 1 (interpersonal) and level 2 (institutional). These articles similarly dealt with medical ethics, but in these instances looked beyond the doctor-patient or researcher-subject relationship to consider the role of involved institutions, whether health care, academic, professional or civil society. Nine of these looked at medical education, and were typified by ethical analysis of short-term educational trips for medical students in HIC going to LMIC. Nine articles examined clinical research with researchers from HIC studying LMIC contexts or issues, and 4 examined humanitarian medical volunteering. Four articles were coded as both level 1 and 3. These articles retained focus on medical research and education, but considered the rights of patients and duties of doctors or researchers vis-a-vis state relationships and international relations.

#### Institutional

Fifteen articles were coded as level 2 (institutional). These articles applied an institutional lens to the analysis of ethical issues germane to industry, health systems, medical education, philanthropic bodies and research programs. Another 17 articles were labelled both levels 2 and 3 (international). These articles dealt with content ranging from medical research, health system resource allocation, infectious disease control, and global health project funding, all from the particular perspective of national governments interacting with allied institutions through international partnerships and/or aid programs.

#### International

Nineteen articles were coded as level 3 (international). The majority of these articles [12] examined international relations and global health governance, looking especially at funding commitments and flows, intergovernmental partnerships, and the constitution, mandate and activities of governments with respect to international organizations. The other topics included resource allocation on an international scale, health worker migration or ‘brain drain’, inter-governmental collaboration for research, and infectious disease pandemic control from the perspective of national government collaboration.

#### Structural

Thirty-three articles were coded as level 4 (structural). Articles in this category examined a broad range of content issues, but all with a focus on phenomena that transcend interstate relations. They tended to focus on system-level issues, including governance and structural concerns, rather than deal with concrete issues and solutions. These issues include the environment and climate change, global pandemic preparedness, global governance, global power dynamics in health, and economic globalization. Seventeen articles were coded as levels 3 (international) and 4, most of which looked at global governance from both the national government perspective and from the global citizenship perspective. This category also included articles on physician migration, national health research projects, and food security from national and global perspectives.

The remaining articles were coded as a combination of three or more of levels. Many of these were reviews looking at multiple issues or comparing and contrasting different ethical frameworks, so did not restrict themselves to one level of analysis.

### Ethical frameworks

The range in conceptual and operational uses of ‘global health ethics’ used were noted as functions of the different disciplines contributing to this body of literature, and their scholarly tools and interests (medicine, bioethics, philosophy, political science, human rights, sociology, anthropology, economics and public health). Most (89%) of the articles concerned with ethics in practice and normative issues were issued from the medical community; many of these were preoccupied with the roles and duties of specific actors in relationships and systems of care. Articles applying specific ethical frameworks tended to be from ethics and philosophy journals, drawing on discourse and methods from moral philosophy and the social sciences. The lines between disciplinary domains were sometimes blurry, but the very fact of contribution from varied schools of thought produced a breadth of approaches, from narrowly practical to deeply theoretical. The ethical frameworks animating the literature encompassed by our review, thus corresponded to four functional categories: those examining practical or narrowly applied ethical questions on health issues with widespread global relevance; those concerned with normative ethics; those examining an issue through a single philosophical tradition; and those comparing and contrasting insights from multiple ethical frameworks.

The level of engagement also corresponded in some cases to the ethical framework used. The majority of articles in level 1 (interpersonal) looked at practical ethical issues occasioned by interpersonal relationships, commonly doctor/patient or researcher/participant. Those that used an explicit ethical framework either applied classic medical ethical principles (autonomy, beneficence, nonmaleficence and justice) to dynamics of care in a global health context, or analyzed relationships of care through a social justice lens. Articles in level 2 (institutional) were typified by attention to practical ethical issues or to the application of normative principles to institutional landscapes – for instance, looking at institutional drivers of inequitable access to healthcare in a ‘global’ context. Level 3 (international) included more articles with explicit philosophical frames, including application of a Rawlsian conception of justice, Peter Singer’s utilitarianism, and Thomas Pogge on cosmopolitanism, as well as human rights and equity-based framings. Jennifer Prah Ruger’s theory of ‘provincial globalism,’ founded in Aristotelian notions of justice and elaborating on Amartya Sen’s capability theory, was acknowledged and critiqued by three response articles dealing with global health governance [13]. Level 4 (structural) was characterized by scholarship leveraging theories of cosmopolitanism and global justice, and invoking notions of global citizenship to argue for universal rights and healthcare access. Several articles from across all levels explored their respective topics from the perspective of varied philosophical traditions, or compared and contrasted distinct ethical frameworks.

## Discussion

### Who can speak?

Perhaps the most morally troubling finding of this review is the lack of voices from the global South in the literature we identified as addressing global health ethics. While this is not wholly surprising, given that our search was limited to English language sources, it does speak to a marginalization of voices from the global south within this discursive space. It is not our intent to speculate on why this might be, for the reasons are no doubt complex and can range from linguistic to epistemological to economic and beyond. They could also include our own limitations in how we designed the study. However, there is a disturbing parallel between disparities in global health, and this discursive disparity in the global health ethics in the English language. While we have not conducted a formal discourse analysis, this does raise the question of how authors from the global South are rendered largely silent in this discursive space, and what power dynamics are at play. We suggest that this issue is of paramount importance to the field, and its very legitimacy as a field of moral scholarship is in question. We need to find a way to include the very people impacted by disparities in global health in conversations about what is owed to them, how to rectify these inequities, and how to study ethical problems in global health in ways that contribute to people’s ability to resist oppression and rectify the kinds of epistemological injustices that may have led to exclusion from this discursive space. For as things stand now, HIC voices dominate the English language conversation on global health ethics, and this is morally problematic if only because it mirrors the very inequities scholars are trying to redress.

### Defining Global Health

It is clear that the term ‘global health ethics’ encompasses issues and relationships that range from micro-level interactions to the supra-national, structural level. This is indicative of the term’s contested meanings: those who use the term descriptively, to indicate geographical location of interactions, are at odds with those who believe the term has, or ought to have, moral content. Foucault would suggest that this is suggestive of some kind of discursive power struggle, and those of us who would like to see it connote a field concerned with structural-level moral analysis clearly have work to do. We believe that work starts with a critical reflection on how differing conceptions of global health *itself* might underlie the paradigmatic and substantive diversity evident in our findings. We found that a clear definition of ‘global health’ was rarely explicitly stated in the literature we reviewed. However, some overarching trends emerged from those articles that did clarify the ambit of, and context for, the term’s use.

In 2009 Koplan et al. (within the Consortium of Universities for Global Health - Table 3) defined global health ‘*as an area for study, research, and practice that places a priority on improving health and achieving equity in health for all people worldwide. Global health emphasises transnational health issues, determinants, and solutions; involves many disciplines within and beyond the health sciences and promotes inter-disciplinary collaboration; and is a synthesis of population-based prevention with individual-level clinical care’*. This definition was one of the most commonly referenced by articles written after 2009, many of which emphasized its distinction from earlier paradigms of ‘international health’ [14].

**Table 3:** Global and International Health: What are the differences?

	Global Health	International Health
Geography	Focuses on issues that transcend national boundaries	Focuses on issues of countries other than one’s own, especially on those of LMIC
Cooperation	Solutions often require global cooperation	Solutions require bi-national cooperation
Objective	Health equity for all peoples, regardless of nationality	Seeks to help those in other countries, particularly through aid going from HIC to LIC
Scope	Inter and multi-disciplinary – beyond the health sciences; including issues directly or indirectly affecting health	May overlap several disciplines but not multi-disciplinary; focus on health issues

Beaglehole and Bonita commented on this definition as being useful (with its broad focus on health improvement and health equity), but also as ‘wordy and uninspiring’. They were critical, too, of the Kickbush definition… *‘those health issues that transcend national boundaries and governments and call for actions on the global forces that determine the health of people’* as also ‘having a broad focus but no clear goal, passive in its call for action, and neglectful of the need for collaboration and research’. Instead they proposed a definition for global health as ‘*collaborative trans-national research and action for promoting health for all’* with the advantage of being shorter and sharper, with an emphasis on the critical need for collaboration, and action orientation [12].

Yet another commonly cited definition, with some overlapping themes, came from Benatar (2013):

*‘Global Health … goes beyond international health to include acknowledgment of health in more than merely a biomedical sense, and the critical interdependence of the health of all in a world characterized, inter-alia by excessive (often wasteful) consumption of limited resources, population growth, demographic changes, porous borders and environmental and biological dangers that threaten all lives globally.’* [15]. This definition is based on the notion that global health should refer to a much more complex notion of population health: one that includes acknowledgment of the social and societal determinants of health, the lack of geographic or social barriers to the spread of infectious diseases; and the importance of the interconnectedness and interdependence of all life and human well-being on an ecologically threatened planet. As such, global health is concerned with health in a world characterized by socially structured economic, communication and other systems forces that have powerful positive and negative impacts on health. This definition, which is the one we prefer, focuses on what we identified as the “structural” level of engagement, arguing that obligations between individuals and species are at least partially rooted in structural arrangements that render particular individuals complicit in global health inequities. It is the only definition that attempts to orient our moral gaze primarily towards the structural level.

Even before 2009, strains of an expansive and contested understanding of ‘global health’ are evident, often encompassed by the term ‘global public health’. However, the majority of articles earlier in our selection, from 1977 to 2009, used the term ‘global health’ to refer to issues and approaches that would fit under Koplan’s definition of ‘international health’. Despite an increasingly well-established distinction between ‘global’ and ‘international’ health, many articles written in the later 2000s and 2010s continued to blur lines between the two, commonly employing the term ‘global health’ to refer to issues that would fall within the domain of ‘international health’. Most of these articles were focused on medical education and research, and used ‘global health’ as a keyword to denote research or clinical care in the global south, rather than the more nuanced and broader conception of ‘global health’ implied in some of the above definitions.

This increasing use of the term ‘global health’ without a meaningful exploration of what the term itself encompasses might relate in part to the political nature of language. ‘New’ is reflexively perceived as better, the vanguard often valued in itself. As the notion of ‘global health’ emerged and vied with ‘international health’ in academic discourse, it attracted prestige and capital – in the form of research funding, institutional investment, and publication cache. We speculate that the dynamics of its use relate not only (or even mostly) to the intellectual dissection of ‘global health’ from ‘international health’, but also to the cyclical interplay between use and impact attached to the normative influence of language and ideas as dominated by the ‘powerful’ [16].

The implications of this phenomenon for making sense of the literature on global health ethics are considerable – indeed foundational. Notionally divergent starting points for ‘global health’ are apt to condition very different understandings of the ethical issues attached to it. Academic endeavors that construe global health as an outgrowth, or lexical recasting, of international health will tend to recapitulate the normative issues that characterized dominant paradigms of scholarship on international health which often reify traditional bioethical concerns that are focused at the level of individual moral relationships, or on organizational ethics. On the other hand, those that identify something essentially distinct in global health are more sensitive to and emphasize the ethical considerations occasioned by that difference. For instance, a definition of global health that extends beyond nation-state dynamics to supranational determinants of health – such as global capital flows or environmental pressures – will focus on what unites and divides us within these encompassing systems, and on solutions that attend to those divisions. The normative focus is thus different, and the attendant obligations are rooted in fundamentally different considerations. This does not imply that nation-states have no moral obligations in addressing global health challenges, rather that the ‘animal’ of nation-state cannot solve them entirely within the context of political and economic globalization driven by the self-interests of the most powerful.

Relatedly, the term ‘global health ethics’ might obscure as much as it illuminates. A limitation of our study relates to the inherent constraints of literature searches, insofar as they are predicated on, and bound by, the language used to define a field. The self-identification of various strains of ethical inquiry in global health as exercises in ‘global health ethics’ has grown in tandem with the field, and in response to the proliferation of global health scholarship itself. But many forms of inquiry germane to the ethical dimensions of global health are not captured through explicit reference to ‘global health ethics’ per se. Mature traditions of scholarship in political economy and political philosophy are just two examples. While the purpose of our work was not to suggest an ideal conception of the field, rather to capture and critique, it is critical to acknowledge that the current language being used in the global north to set it apart might impoverish rather than enhance it, as compared with work contributed from the global south that could set the frame for enriching the debate and encouraging new paradigms of thinking and action [10, 11, 15, 17].

## Conclusion

The purpose of this review is not to argue for a particular normative frame for global health ethics, but rather to raise critical issues that bear on the normative direction of this emerging field. We do not claim that identification with the field by any specific tranche of the literature is illegitimate; nor do we offer a unifying definition for the field. This Critical Interpretive Review is intended to serve as the first step in an ongoing deliberative process that seeks to better delineate the scope and charge of global health ethics scholarship and hopefully to develop a normative concept responsive to truly global needs and health trends. As the term ‘global health ethics’ is still a contested one, about which no one ‘truth’ has yet been constructed, we see this as a moment (or a snapshot) in a broader discursive process in which language reflects and both de-constructs and re-constructs this relatively young area of scholarship. We hope that this overview of the contours of the field will help to map the normative way forward as we grapple with the ethical issues in global and planetary health. Going forward, two common and critical themes in the literature should be heeded. First is the need to acknowledge the challenges of extraordinary disparities in health, with inability to narrow these adequately merely through the now discredited idea of economic growth and trickle-down effect. This entails serious moral consideration of what we have termed the structural level of engagement in global health. Second is the need to afford the global south a stronger voice in any such debate [17], in order to constructively include consideration of alternative ontologies, epistemologies and moral and political values in seeking solutions to seemingly intractable problems.

## Additional file

Additional file 1: Supplementary Appendix Articles Reviewed.docx. This file contains a list of all the articles reviewed for this study. (DOCX 44 kb)

## Data Availability

All data generated or analyzed during this study are included as a Additional file 1.

## Abbreviations

HIC: High-income countries
LMIC: Low- or middle-income countries
