# Supplementary material for: Global health ethics: critical reflections on the contours of an emerging field, 1977–2015
Source: BMC Med Ethics. 2019 Jul 25;20:53. doi: 10.1186/s12910-019-0391-9 (PMC6657376; doi:10.1186/s12910-019-0391-9)
Supplement: Supplementary file 1 — Supplementary Appendix Articles Reviewed.docx. This file contains a list of all the articles reviewed for this study. (DOCX 44 kb) [file 12910_2019_391_MOESM1_ESM.docx]

**Appendix 1: Critical Interpretive Review Articles**

| Alkire, S., and Chen, L. (2004). ‘Global Health and Moral Values.’ *The Lancet* 364:1069–74. |
| --- |
| Asgary, R., and Junck, E. (2013). ‘New Trends of Short-Term Humanitarian Medical Volunteerism: Professional and Ethical Considerations.’ *Journal of Medical Ethics* 39:625–31. |
| Barnard, D., Bui, T. T., Chase, J., Jones, E., Loeliger, S., Velji, A., and White, M. T. (2011). ‘Ethical Issues in Global Health Education.’ *Global Health Training in Graduate Medical Education: A Guidebook*, 25-43. |
| Benatar, D. (2011). ‘Animals, the environment and global health’ in S. Benatar, and G. Brock (Ed.), *Global Health and Global Health Ethics* (210-220). Cambridge, UK: Cambridge University Press. |
| Benatar, S. (1998). ‘Imperialism, Research Ethics and Global Health’. *Journal of Medical Ethics* 24:221–22. |
| Benatar, S. (2011). ‘Global Leadership, Ethics and Global Health: The Search for New Paradigms’. in S. Gill (Ed.), *Global Crises and the Crisis of Global Leadership* (127–143). Cambridge: Cambridge University Press. |
| Benatar, S. (2013). ‘Global Health and Justice: Re-Examining Our Values.’ *Bioethics* 27(6):297–304. |
| Benatar, S. (2015). ‘Explaining and Responding to the Ebola Epidemic.’ *Philosophy, Ethics, and Humanities in Medicine* 10:5. |
| Benatar, S., and Brock, G. (2011)*.* ‘Introduction’ in S. Benatar, and G. Brock (Ed.), *Global Health and Global Health Ethics* (1-10). Cambridge, UK: Cambridge University Press. |
| Benatar, S., and Upshur, R. (2014). ‘Virtues and Values in Medicine Revisited: Individual and Global Health.’ *Journal of the Royal College of Physicians of London: Clinical Medicine* 14(5):495-499. |
| Benatar, S., Daar, A., and Singer, P. (2003). ‘Global Health Ethics: The Rationale for Mutual Caring.’ *International Affairs* 79(1):107–38. |
| Benatar, S., Gill, S., and Bakker, I. (2009). ‘Making Progress in Global Health: The Need for New Paradigms.’ *International Affairs* 85(2):347–371. |
| Bennett, G. (2013). ‘H5N1 and the Politics of Truth.’ *Hastings Center Report* 43(2):35–37. |
| Bettcher, D., and Yach, D. (1998). ‘The Globalisation of Public Health Ethics?’ Millennium. *Journal of International Studies* 27(3):469–96. |
| Bhutta, Z. A. (2002). ‘Ethics in International Health Research: A Perspective from the Developing World.’ *Bulletin of the World Health Organization* 80(2):114–20. |
| Birn, A. E. (2011). ‘Addressing the societal determinants of health: the key global health ethics imperative of our times’ in S. Benatar, and G. Brock (Ed.), *Global Health and Global Health Ethics* (37-52). Cambridge, UK: Cambridge University Press. |
| Brock, G. (2012). ‘Global Health and Responsibility’ in Health Inequalities and Global Justice. Edinburgh: Edinburgh University Press. |
| Brock, G. (2011). ‘International taxation’ in S. Benatar, and G. Brock (Ed.), *Global Health and Global Health Ethics* (274-284). Cambridge, UK: Cambridge University Press. |
| Brown, G. (2012). ‘Re-examining the Ethical foundations: behind the distribution of global health’ in *Health Inequalities and Global Justice*. Edinburgh: Edinburgh University Press. |
| Brownsword, R. (2015). ‘Patents and intellectual property rights’ in H. Widdows, and D. Moellendorf (Eds.), *The Routledge Handbook of Global Ethics*. Abingdon, Oxon: Routledge. |
| Buchanan, A., and Decamp, M. (2006). ‘Responsibility for Global Health.’ *Theoretical Medicine and Bioethics* 27:95–114. |
| Caris,G., and Namboya, F. (2015). ‘Ethics of Global Health Care.’ *International Anesthesiology Clinics* 53(3):90–97. |
| Cole et al. (2013). ‘Teaching Global Health Ethics’ in *An Introduction to Global Health Ethics*. Abingdon, Oxon: Routledge. |
| Crane, J. (2010). ‘Adverse Events and Placebo Effects: African Scientists, HIV, and Ethics in the ‘Global Health Sciences.'’ *Social Studies of Science* 40(6):843–70. |
| Crigger, N. J. (2008). ‘Towards a Viable and Just Global Nursing Ethics.’ *Nursing Ethics* 15(1):17–27. |
| Crump, J. A., and J Sugarman. (2008) ‘Ethical Considerations for Short-Term Experiences by Trainees in Global Health.’ *Journal of the American Medical Association*, 300:1456-1458. |
| Crump, J. A., and Sugarman, J. (2010). ‘Ethics and Best Practice Guidelines for Training Experiences in Global Health.’ *The American Journal of Tropical Medicine and Hygiene* 83(6):1178–82. |
| Dacso, M., Chandra, M., and Friedman, H. (2013). ‘Adopting an Ethical Approach to Global Health Training: The Evolution of the Botswana-University of Pennsylvania Partnership.’ *Academic Medicine* 88(11):1646-1650. |
| Daniels, N. (2011). ‘International health inequalities and global justice: toward a middle ground’ in S. Benatar, and G. Brock (Ed.), *Global Health and Global Health Ethics* (97-107). Cambridge, UK: Cambridge University Press. |
| Dauda, B., and Dierickx, K. (2012). ‘Health, Human Right, and Health Inequalities: Alternative Concepts in Placing Health Research as Justice for Global Health.’ *The American Journal of Bioethics* 12(11):42–44. |
| DeCamp, et al. (2013). ‘An Ethics Curriculum for Short-Term Global Health Trainees.’ *Globalization and Health* 9:5. |
| DeCamp, M. (2011). ‘Ethical Review of Global Short-Term Medical Volunteerism.’ *Journal on Hospitals* 23:91-103. |
| Degeling, C., Mayes, C., Lipworth W., Kerridge, I., qnd Upshur, R. (2015). ‘The Political and Ethical Challenge of Multi-Drug Resistant Tuberculosis.’ *Journal of Bioethical Inquiry* 12:107–13. doi:10.1007/s11673-014-9595-3. |
| Dell, E. M., Varpio, L., Petrosoniak, A., Gajaria, A., & McMcarthy, A. E. (2014). ‘The Ethics and Safety of Medical Student Global Health Electives.’ *Journal of Medical Education* 5:63-72. doi:10.5116/ijme.5334.8051. |
| Denburg, A. E. (2010). ‘Global Child Health Ethics: Testing the Limits of Moral Communities.’ *Public Health Ethics* 3(3):239–58. |
| Donovan, K. G. (2014). ‘Ebola, Epidemics, and Ethics - What We Have Learned.’ *Philosophy, Ethics, & Humanities In Medicine* 9:15. |
| Doomen, J. (2012) ‘Distributing Health.’ *American Journal of Bioethics* 12(12):63. |
| Dwyer, J. (2005). ‘Global health and justice.’ *Bioethics* 19(5‐6):460-475. |
| Dwyer, J. (2009). ‘How to Connect Bioethics and Environmental Ethics: Health, Sustainability, and Justice.’ Bioethics 23(9):497–502. |
| Dwyer, J. (2011).’Teaching global health ethics’ in S. Benatar, and G. Brock (Ed.), *Global Health and Global Health Ethics* (319-328). Cambridge, UK: Cambridge University Press. |
| Eckenweiler, L. (2012). ‘Ecological Subjects, Ethical Placemaking and Global Health Equity’ in *Health Inequalities and Global Justice*. Edinburgh: Edinburgh University Press. |
| Eckenwiler, L., and Matthew H. (2014). ‘Counterterrorism, Ethics, and Global Health.’ *Hastings Center Report* 44(3):12–13. |
| Eckenwiler, L., Straehle, C., and Chung, R. (2012). ‘Global Solidarity, Migration and Global Health Inequity.’ Bioethics 26(7):382–390. |
| Fitchett, J. R. (2009). ‘Ethical Considerations of Clinical Trials in the Developing World.’ Transactions of the Royal Society of Tropical Medicine and Hygiene 103:756-760. |
| Forman, L., and Nixon, S. (2013). ‘Human Rights Discourse in Global Health’ in *An Introduction to Global Health Ethics.* Abingdon, Oxon: Routledge. |
| Friel, S., Butler, C., and McMichael, A. (2011). ‘Climate change and health: risks and inequities’ in S. Benatar, and G. Brock (Ed.), *Global Health and Global Health Ethics* (198-209). Cambridge, UK: Cambridge University Press. |
| Gibson, J., Forman, L., and Nixon, S. (2015). ‘Editorial: Bioethics and the Right to Health: Advancing a Complementary Agenda.’ *Health & Human Rights* 17(1):1-5. |
| Glover, J. (2011). ‘Poverty, distance and two dimensions of ethics’ in S. Benatar, and G. Brock (Ed.), *Global Health and Global Health Ethics* (311-318). Cambridge, UK: Cambridge University Press. |
| Gostin, L. (2015). ‘Good Science + Good Ethics = Good Law: Five Rules for Epidemic Preparedness.’ *Milbank Quarterly* 93(1):19–23. |
| Gostin, L., and Mok, E. (2010). ‘Innovative Solutions to Closing the Health Gap between Rich and Poor: A Special Symposium on Global Health Governance.’ *Journal of Law, Medicine & Ethics* 38(3):451–58. |
| Gostin, L.., and Hodge, J. (2007). ‘Global Health Law, Ethics, and Policy.’ *Journal of Law, Medicine & Ethics 35(4):519-525.* |
| Haker, H. (2015). ‘Reproductive Rights and reproductive technologies’ in H. Widdows, and D. Moellendorf (Eds.), *The Routledge Handbook of Global Ethics*. Abingdon, Oxon: Routledge. |
| Hall, A. (2006). ‘Whose Progress? The Language of Global Health.’ *Journal of Medicine and Philosophy 31:285-*304. |
| Harmon, S. (2006). ‘Solidarity: A (New) Ethic for Global Health Policy.’ *Health Care Analysis* 14(4):215–36. |
| Harrowing, J., Mill, J., Spiers, J., Kulig, J., and W Kipp. (2010). ‘Culture, Context and Community: Ethical Considerations for Global Nursing Research.’ *International Nursing Review 57:70-77*. |
| Have, T., Henk A. (2011). ‘Global Bioethics and Communitarianism.’ *Theoretical Medicine and Bioethics* 32(5):315–26. |
| Heavey, P. (2012). ‘Global Health Justice and Governance for Synthetic Biology.’ *American Journal of Bioethics* 12(12):64–65. |
| Hirshon, J., Hansoti, B., Hauswald, M., Sethuraman, K., Kerr, N., Scordino, D., and Biros, M. (2013). ‘Ethics in Acute Care Research: A Global Perspective and Research Agenda.’ *Academic Emergency Medicine* 20(12):1251–58. |
| Hughes, S., and Jandial, R. (2013). ‘Ethical Considerations in Targeted Paediatric Neurosurgery Missions.’ *Journal of Medical Ethics* 39(1):51–54. |
| Huish, R. (2012). ‘The Ethical Conundrum of International Health Electives in Medical Education.’ *Journal of Global Citizenship & Equity Education* 2(1). |
| Huish, R. (2009). ‘How Cuba’s Latin American School of Medicine Challenges the Ethics of Physician Migration.’ *Social Science & Medicine* 69(3):301–304. |
| Hunt, M., and Godard, B. (2013). ‘Beyond Procedural Ethics: Foregrounding Questions of Justice in Global Health Research Ethics Training for Students.’ *Global Public Health* 8(6):713-724. |
| Hunt, M., Schwartz, L., Sinding, C., and Elit, L. (2014). ‘The Ethics of Engaged Presence: A Framework for Health Professionals in Humanitarian Assistance and Development Work.’ *Developing World Bioethics* 14(1):47–55*.* |
| Hunter, D., and Dawson, A. (2011). ‘Is there a need for global health ethics? For and against’ in S. Benatar, and G. Brock (Ed.), *Global Health and Global Health Ethics* (77-88). Cambridge, UK: Cambridge University Press. |
| Hurst, S., Mezger, N., and Mauron, A. (2011). ‘Allocating resources in humanitarian medicine’ in S. Benatar, and G. Brock (Ed.), *Global Health and Global Health Ethics* (173-183). Cambridge, UK: Cambridge University Press. |
| Hussein, M., and Upshur, R. (2013). ‘Ethical challenges in global health research’ in *An Introduction to Global Health Ethics*. Abingdon, Oxon: Routledge. |
| IJsselmuiden, C. (2010). ‘Evolving Values in Ethics and Global Health Research’*Global Public Health* 5(2):154–163. |
| Illingworth, P., and Parmet, W. (2012). ‘Solidarity for Global Health: Editorial.’ *Bioethics* 26(7):ii – iv. |
| Johri, M., Chung, R., Dawson, A., and Schrecker, T. (2012). ‘Global Health and National Borders: The Ethics of Foreign Aid in a Time of Financial Crisis.’ *Globalization and Health* 8:19. |
| Kaida, A., and Lenard, P. (2012). ‘Outlining the global duties of justice owed to women living with HIV/AIDS in SSA’ in *Health Inequalities and Global Justice*. Edinburgh: Edinburgh University Press. |
| Kass, N. (2004). ‘Public Health Ethics: From Foundations and Frameworks to Justice and Global Public Health.’ *The Journal of Law, Medicine & Ethics* 32(2):232–42. |
| Katz, C, Lahey, T., and Campbell, H. (2014). ‘An Ethical Framework for Global Psychiatry.’ Annals of Global Health 80(2):146–51. |
| Kiddell-Monroe, R. (2014). ‘Access to Medicines and Distributive Justice: Breaching Doha’s Ethical Threshold: Breaching Doha’s Ethical Threshold.’ *Developing World Bioethics* 14(2):59–66. |
| Kirby, K., and Siplon, P. (2012). ‘Push, Pull, and Reverse: Self-Interest, Responsibility, and the Global Health Care Worker Shortage.’ *Journal of Health Philosophy and Policy* 20:152–176*.* |
| Kiromena, Philpott, Marsh, and Chan. (2013).’Ethics and Clinical Medicine’ in *An Introduction to Global Health Ethics*. Abingdon, Oxon: Routledge. |
| Koivusalo, M. (2011). ‘Trade and health: the ethics of global rights, regulation and redistribution’ in S. Benatar, and G. Brock (Ed.), *Global Health and Global Health Ethics* (143-154). Cambridge, UK: Cambridge University Press. |
| Labonte, R. (2014). ‘Health in All (Foreign) Policy: Challenges in Achieving Coherence.’ *Health Promotion International* 29:S1*.* |
| Labonte, R., and Gagnon, M. (2010). ‘Framing Health and Foreign Policy: Lessons for Global Health Diplomacy.’ *Globalization and Health* 6:14. |
| Labonte, R., and Schrecker, T. (2011). ‘The state of global health in a radically unequal world: patterns and prospects’ in S. Benatar, and G. Brock (Ed.), *Global Health and Global Health Ethics* (24-36). Cambridge, UK: Cambridge University Press. |
| Lairumbi, G., Michael, P., Fitzpatrick, R., and English, M. (2011). ‘Ethics in Practice: The State of the Debate on Promoting the Social Value of Global Health Research in Resource Poor Settings Particularly Africa.’ *BMC Medical Ethics* 12(1):22. |
| Langowski, M., and Iltis, A. (2011). ‘Global Health Needs and the Short-Term Medical Volunteer: Ethical Considerations.’ *HealthCare Ethics Committee Forum* 23(2):71–78. |
| Lassen, L., and Thomsen, M. (2007). ‘Global health--the ethical responsibility of the pharmaceutical industry.’ *Dan Med Bull* 54(1):35-6. |
| Lavery, J., Green, S., Bandewar, S., Bhan, A., Daar, A., Emerson, E., Masum, H., Randazzo, F., Singh, J., and Singer, P. (2013). ‘Addressing Ethical, Social, and Cultural Issues in Global Health Research.’ D. Diemert (Ed.). *PLOS Neglected Tropical Disease*s 7(8):e2227. |
| Lavery, J., Upshur, R., Sharp, R., and Hofman, K. (2003). ‘Ethical Issues in International Environmental Health Research.’ *International Journal of Hygiene and Environmental Health* 206:453-463. |
| Lee, Bandy X, and John L Young. (2012). ‘Building a Global Health Ethic without Doing Further Violence.’ *American Journal of Bioethics* 12(12):59–60. |
| Lencucha, R. (2013). ‘Cosmopolitanism and Foreign Policy for Health: Ethics for and beyond the State.’ *BMC International Health and Human Rights* 13(1):29. |
| Levine, C. (1977). ‘Ethics, Justice, and International Health.’ *The Hastings Center Repor*t 7(2):5–7. |
| Lindauer, M. (2012). ‘The Focus on Health Capability and Role of States in Ruger’s Global Health Justice Framework.’ *American Journal of Bioethics* 12(12):57–59. |
| Logar, T., Le, P., Harrison, J., and Glass, M. (2015). ‘Teaching Corner: ‘First Do No Harm’: Teaching Global Health Ethics to Medical Trainees Through Experiential Learning.’ *Journal of Bioethical Inquiry* 12(1):69–78. |
| London, A. (2011).’Justice and research in developing countries’ in S. Benatar, and G. Brock (Ed.), *Global Health and Global Health Ethics* (293-303). Cambridge, UK: Cambridge University Press. |
| London, L. (2008). ‘What Is a Human Rights-Based Approach to Health and Does It Matter?’ *Health and Human Rights* 10(1):65-80. |
| Lowry, C. (2009). ‘Two Models in Global Health Ethics.’ *Public Health Ethics* 2(3):276–284. |
| Mackey, T., and Liang, B. (2012). ‘Integrating Biodiversity Management and Indigenous Biopiracy Protection to Promote Environmental Justice and Global Health.’ *Journal of Public Health* 102(6):1091-1095. |
| Macklin, R. (2012). ‘Ethics in Global Health: Research, Policy and Practice.’ *OUP USA.* New York: Oxford University Press. |
| Macklin, R. (2012). ‘Good in Theory: Can It Work in Practice?’ *American Journal of Bioethics* 12(12):55–56. |
| Macpherson, C. (2014). ‘Climate Change Matters.’ *Journal of Medical Ethics* 40(4):288–290. |
| Martin, B., Love, T., Srinivasan, J., Sharma, J., Pettitt, B., Sullivan, C., Pattaras, J., Master, V., and Brewster, L. (2014). ‘Designing an Ethics Curriculum to Support Global Health Experiences in Surgery.’ *Journal of Surgical Research* 187:367-370*.* |
| Masum, H., Chakma, J., and Daar, A. (2011).’Biotechnology and global health’ in S. Benatar, and G. Brock (Ed.), *Global Health and Global Health Ethics* (251-260). Cambridge, UK: Cambridge University Press. |
| McCall, D., and Iltis, A. (2014), ‘Health Care Voluntourism: Addressing Ethical Concerns of Undergraduate Student Participation in Global Health Volunteer Work.’ *HealthCare Ethics Committee Forum* 26(4):285–97. |
| McIntyre, L., and Rondeau, K. (2011).’Food security and global health’ in S. Benatar, and G. Brock (Ed.), *Global Health and Global Health Ethics* (261-273). Cambridge, UK: Cambridge University Press. |
| Meier, B., and Fox, A. (2010). ‘International Obligations through Collective Rights: Moving from Foreign Health Assistance to Global Health Governance.’ *Health and Human Rights* 12(1):61-72. |
| Merritt, M. (2007). ‘Bioethics, Philosophy, and Global Health.’ *Yale Journal of Health Policy, Law, and Ethics* 7(2):273-317. |
| Muntaner, C., Ng, E., and Chung, H. (2012). ‘Making Power Visible in Global Health Governance.’ *The American Journal of Bioethics* 12(7):63–64. |
| Murphy et al. (2013). ‘Ethical considerstaions of global health partnerships’ in *An Introduction to Global Health Ethics*. Abingdon, Oxon: Routledge. |
| Murphy, J., Hatfield, J., Afsana, K., and Neufeld, V. (2015). ‘Making a Commitment to Ethics in Global Health Research Partnerships: A Practical Tool to Support Ethical Practice.’ *Journal of Bioethical Inquiry* 12(1):137–46. |
| Myser, C. (2015). ‘Defining ‘Global Health Ethics.’’ *Journal of Bioethical Inquiry* 12(1):5–10. |
| Naidoo, S., and Vernillo, A. (2014). ‘Adapting a Community of Practice Model to Design an Innovative Ethics Curriculum in Healthcare.’ *Medical Principles and Practice* 23(1):60-68. |
| Niekerk, A. (2004). ‘Principles of Global Distributive Justice: Moving beyond Rawls and Buchanan.’ *South African Journal of Philosophy* 23(2):171–194. |
| Nixon, S. (2006). ‘Critical Public Health Ethics and Canada’s Role in Global Health.’ *Canadian Journal of Public Health* 97(1):32–34. |
| Nixon, S., and Forman, L. (2008). ‘Exploring Synergies between Human Rights and Public Health Ethics: A Whole Greater than the Sum of Its Parts.’ *BMC International Health and Human Rights* 8:2. |
| Olusanya, B. (2008). ‘Global Health Priorities for Developing Countries: Some Equity and Ethical Considerations.’ *Journal of the National Medical Association* 100(10):1212–1217. |
| Ooms, G., and Hammonds, R. (2010). ‘Taking up Daniels’ Challenge: The Case for Global Health Justice.’ *Health and Human Rights* 12(1):29-46. |
| Oprea, L., Braunack-Mayer, A., and Gericke, C. (2009). ‘Ethical Issues in Funding Research and Development of Drugs for Neglected Tropical Diseases.’ *Journal of Medical Ethics* 35:310–314. |
| Ozeren, G., and Cabar, H. (2013). ‘Reflections of Globalization and Health Informatics on Medical Ethics.’ *Journal of Experimental and Clinical Medicine* 30:99-102. |
| Pang, T. (2011).’Global health research: changing the agenda’ in S. Benatar, and G. Brock (Ed.), *Global Health and Global Health Ethics* (285-292). Cambridge, UK: Cambridge University Press. |
| Parker, M., and Allen, T. (2013). ‘Questioning Ethics in Global Health’ in *Ethics in the Field : Contemporary Challenges* (24-41). New York: Berghahn Books. |
| Patz, J., Gibbs, H., Foley, J., Rogers, J., and Smith, K. ( 2007). ‘Climate Change and Global Health: Quantifying a Growing Ethical Crisis.’ *EcoHealth* 4(4):397–405. |
| Pettus, K. I. (2012). ‘Global Govern-Mentality?’ *American Journal of Bioethics* 12(12):61–62. |
| Phillips, W. and Rathert, C. (2012). ‘Health Ethics in a GLobal Context; Ethical Models and Applications of Globalization: Cultural, Socio-Political and Economic Perspectives.’ in C. Wankel, and S. Malleck (Ed.), *IGI Global* (100-118). |
| Pinto, A., and Smylie, J. (2013). ‘Indigenous health and ethics’ in *An Introduction to Global Health Ethics.* Abingdon, Oxon: Routledge. |
| Pinto, A., and Upshur, R. (2009). ‘Global Health Ethics for Students.’ *Developing World Bioethics* 9(1):1–10. |
| Pinto, A., Birn, A. E., and Upshur, R. (2013). ‘Context of Global Health Ethics’ in *An Introduction to Global Health Ethics*. Abingdon, Oxon: Routledge. |
| Pogge, T (2011).’The Health impact fund: how to make new medicines accessible to all’ in S. Benatar, and G. Brock (Ed.), *Global Health and Global Health Ethics* (241-250). Cambridge, UK: Cambridge University Press. |
| Pogge, T. (2005). ‘Human Rights and Global Health: A Research Program.’ *Metaphilosophy* 36(1-2): 182–209. |
| Pratt, B., and Hyder, A. (2015). ‘Applying a Global Justice Lens to Health Systems Research Ethics: An Initial Exploration.’ *Kennedy Institute of Ethics Journal* 25(1):35–66. |
| Pratt, B., and Hyder, A. (2015). ‘Global Justice and Health Systems Research in Low- and Middle-Income Countries.’ *The Journal of Law, Medicine & Ethics* 43(1):143–161. |
| Pratt, B., and Loff, B. (2011). ‘Justice in International Clinical Research.’ *Developing World Bioethics* 11(2):75–81. |
| Pratt, B., and Loff, B. (2013). ‘Linking International Research to Global Health Equity: The Limited Contributino of Bioethics.’ *Bioethics* 27(4):208–214. |
| Pratt, B., and Loff, B. (2014). ‘A Framework to Link International Clinical Research to the Promotion of Justice in Global Health.’ *Bioethics* 28(8):387–96. |
| Pratt, B., Zion, D., and Loff, B. (2012). ‘Evaluating the Capacity of Theories of Justice to Serve as a Justice Framework for International Clinical Research.’ *American Journal of Bioethics* 12(11):30–41. |
| Pratt, B., Zion, D., Lwin, K., Cheah, P., Nosten, F., and Loff, B. (2014). ‘Linking International Clinical Research with Stateless Populations to Justice in Global Health.’ *BMC Medical Ethics* 15(1):49. |
| Provenzano, A., Graber, L., Elansary, M., Khoshnood, K., Rastegar A., and Barry, M. (2010). ‘Short-Term Global Health Research Projects by US Medical Students: Ethical Challenges for Partnerships.’ *Journal of Tropical Medicine and Hygiene* 83(2):211–214. |
| Reisch, R. (2011). ‘International Service Learning Programs: Ethical Issues and Recommendations.’ *Developing World Bioethics* 11(2):93–98. |
| Romo, M., and DeCamp, M. (2015). ‘Ethics in Global Health Outreach: Three Key Considerations for Pharmacists.’ *International* *Journal of Pharmacy Practice* 23:86–89. |
| Roth, D. (2003). ‘An Ethics-Based Approach to Global Child Health Research.’ *Paediatrics & Child Health* 8(2):67. |
| Ruger, J. (2006). ‘Ethics and Governance of Global Health Inequalities.’ *Journal of Epidemiology and Community Health* 60(11):998–1002. |
| Ruger, J. (2007). ‘Rethinking Equal Access: Agency, Quality, and Norms.’ *Global Public Health*  2(1):78-96. |
| Ruger, J. (2009). ‘Global Health Justice.’ *Public Health Ethics,* 1-15. |
| Ruger, J. (2012). ‘Global Health Justice and Governance.’ *The American Journal of Bioethics* 12(12):35–54. |
| Ruger, J. (2015). ‘Good Medical Ethics, Justice and Provincial Globalism.’ *Journal of Medical Ethics* 41(1):103–106. |
| Ruiz-Casares, M. (2014). ‘Research Ethics in Global Mental Health: Advancing Culturally Responsive Mental Health Research.’ *Transcultural Psychiatry* 51(6):790–805. |
| Sanchez, L. (2013). ‘Perspectives on global health form the south’ in *An Introduction to Global Health Ethics*. Abingdon, Oxon: Routledge. |
| Schloss, A., Bavishi, R., Garetto, L., and Shah, M. (2014). ‘Sustainability and the Principle of Respect for Community as a Means of Enhancing Healthcare Equality.’ *Journal of the American College of Dentists* 81(1):31–35. |
| Schrecker, T. (2013). ‘Interrogating Scarcity: How to Think about ‘Resource-Scarce Settings.’’ *Health Policy and Planning* 28:400–409. |
| Schuftan, C. (2009). ‘The Role of Ethics and Ideology in Our Contribution to Global Health.’ *Global Health Action*. |
| Schuklenk, U. (2014). ‘Bioethics and the Ebola Outbreak in West Africa’ *Developing World Bioethics* 14(3):ii – iii. |
| Schuklenk, U., and Cline, C. (2013). ‘Global Health Ethics.’ in H. LaFollette (Ed.), *The International Encyclopedia of Ethics* (2152–2162). |
| Schuklenk, U., and Smalling, R. (2015). ‘Research Ethics’ in H. Widdows, and D. Moellendorf (Eds.), *The Routledge Handbook of Global Ethics*. Abingdon, Oxon: Routledge. |
| Selgelid, M. (2008). ‘Improving Global Health: Counting Reasons Why.’ *Developing World Bioethics* 8(2):115–25. |
| Selgelid, M. (2011). ‘Justice, infectious disease and globalisation’ in S. Benatar, and G. Brock (Ed.), *Global Health and Global Health Ethics* (89-96). Cambridge, UK: Cambridge University Press. |
| Shaw, D., and Rich, L. (2015). ‘Intergenerational Global Heath.’ *Journal of Bioethical Inquiry* 12(1):1–4. |
| Simon, C., and Mosavel, M. (2011). ‘Getting Personal: Ethics and Identity in Global Health Research.’ *Developing World Bioethics* 11(2):82–92. |
| Singer, P., and Benatar, S. (2001). ‘Beyond Helsinki: A Vision for Global Health Ethics.’ *BMJ* 322:747–748. |
| Singer, P., Taylor, A., Daar, A., Upshur, R., Singh, J., and Lavery, J. (2007). ‘Grand Challenges in Global Health: The Ethical, Social and Cultural Program.’ *PLoS Medicine* 4(9):1440-1444. |
| Singh, J. (2013). ‘Global Health Governance and Ethics’ in *An Introduction to Global Health Ethics*. Abingdon, Oxon: Routledge. |
| Smith, E., Hunt, M., and Master, Z. (2014). ‘Authorship Ethics in Global Health Research Partnerships between Researchers from Low or Middle Income Countries and High Income Countries.’ *BMC Medical Ethics* 15(42). |
| Stapleton, G., Schröder-Bäck, P., Laaser, U., Meershoek, A., and Popa, D. (2014). ‘Global Health Ethics: An Introduction to Prominent Theories and Relevant Topics.’ *Global Health Action* 7:23569. |
| Stein, D., and Giordano, J. (2015). ‘Global Mental Health and Neuroethics.’ *BMC Medicine* 13(1):44. |
| Stewart, K., and Sewankambo, N. (2010). ‘Okukkera Ng’omuzungu (lost in Translation): Understanding the Social Value of Global Health Research for HIV/AIDS Research Participants in Uganda.’ Global Public Health 5(2):164-180. |
| Stewart, K., Keusch, G., and Kleinman, A. (2010). ‘Values and Moral Experience in Global Health: Bridging the Local and the Global.’ Global Public Health 5(2):115–21. |
| Stuckler, D., Basu, S., and McKee, M. (2011). ‘Global Health Philanthropy and Institutional Relationships: How Should Conflicts of Interest Be Addressed?’ PLoS Medicine 8(4):1-10. |
| Thompson, A., Smith, M., McDougall, C., Bensimon, C., and Perez, D. (2015). ‘‘With Human Health It’s a Global Thing’: Canadian Perspectives on Ethics in the Global Governance of an Influenza Pandemic.’ Journal of Bioethical Inquiry 12(1):115–27. |
| Tilburt, J., and Kaptchuk, T. (2008). ‘Herbal Medicine Research and Global Health: An Ethical Analysis.’ Bulletin of the World Health Organization 86(8):594-599. |
| Upshur, R., Benatar, S., and Pinto, A. (2013). ‘Ethics and Global Health’ in An Introduction to Global Health Ethics. Abingdon, Oxon: Routledge. |
| Venkatapuram, S. (2012). ‘Health inequalities, capabilities and global justice’ in Health Inequalities and Global Justice. Edinburgh: Edinburgh University Press. |
| Voo, T., and Campbell, A. (2015). ‘Trade in Human Body Parts’ in H. Widdows, and D. Moellendorf (Eds.), The Routledge Handbook of Global Ethics. Abingdon, Oxon: Routledge. |
| White, M., and Evert, J. (2014). ‘Developing Ethical Awareness in Global Health: Four Cases for Medical Educators: Developing Ethical Awareness.’ Developing World Bioethics 14(3):111–116. |
| Wikler, D., and Cash, R. (2009). ‘Ethical Issues in Global Public Health’ in R. Beaglehole, and R. Bonita (Eds.), Global Public Health. |
| Wolff, J. (2011). ‘The human right to health’ in S. Benatar, and G. Brock (Ed.), Global Health and Global Health Ethics (108-118). Cambridge, UK: Cambridge University Press. |
| Wolinsky, H. (2007). ‘Bioethics Goes Global. A Growing Coalition of Scientists, Ethicists and Wealthy Benefactors Is Turning Its Attention to Global Health Problems.’ EMBO Reports 8(6):534-536. |
| Wright, D., Flis, N., and Gupta, M. (2008). ‘The ‘Brain Drain’ of Physicians: Historical Antecedents to an Ethical Debate, C. 1960-79.’ Philosophy, Ethics, and Humanities in Medicine 3(24). |
| Yassi, A., Breilh, J., and Dharamsi, S. (2013). ‘The Ethics of Ethics Reviews in Global Health Research: Case Studies Applying a New Paradigm.’ Journal of Academic Ethics 11(2):83–101. |
